# Transductive Feature Selection Using Clustering-Based Sample Entropy for Temperature Prediction in Weather Forecasting

**DOI:** 10.3390/e20040264

**Published:** 2018-04-10

**Authors:** Zahra Karevan, Johan A. K. Suykens

**Affiliations:** ESAT-STADIUS (Department of Electrical Engineering-Stadius Centre for Dynamical Systems, Signal Processing and Data Analytics), KU Leuven, Kasteelpark Arenberg 10, B-3001 Leuven, Belgium

**Keywords:** transductive learning, conditional entropy, information transfer, feature selection, weather forecasting

## Abstract

Entropy measures have been a major interest of researchers to measure the information content of a dynamical system. One of the well-known methodologies is sample entropy, which is a model-free approach and can be deployed to measure the information transfer in time series. Sample entropy is based on the conditional entropy where a major concern is the number of past delays in the conditional term. In this study, we deploy a lag-specific conditional entropy to identify the informative past values. Moreover, considering the seasonality structure of data, we propose a clustering-based sample entropy to exploit the temporal information. Clustering-based sample entropy is based on the sample entropy definition while considering the clustering information of the training data and the membership of the test point to the clusters. In this study, we utilize the proposed method for transductive feature selection in black-box weather forecasting and conduct the experiments on minimum and maximum temperature prediction in Brussels for 1–6 days ahead. The results reveal that considering the local structure of the data can improve the feature selection performance. In addition, despite the large reduction in the number of features, the performance is competitive with the case of using all features.

## 1. Introduction

Entropy measures have been used for many years to exploit the amount of information that a system contains. They play a significant role in interpreting and describing the dynamics of real-life complex networks such as climate, financial, physiological, Earth and medical systems [1,2,3,4,5,6]. There can be model-based or model-free approaches to evaluate the entropy measures. While model-based approaches benefit from the prior knowledge about the probability distribution of the data, model-free methods estimate the probability distribution based on the data. Since in many real-life applications, the probability distribution of the data is unknown, in this study, we use a model-free approach known as sample entropy, which is one of the popular methods for analyzing the complexity of a dynamical system.

Moreover, in time series analysis, entropy measures can be utilized to illustrate the strength and the direction of causality. The authors in [7] investigate a bivariate dynamical system and suggest conditional entropy to evaluate the amount of information in one particular state of a process when the history of the other one is known. One major concern while using conditional entropy is the number of previous values, known as lag or delay, in the conditioning term. Furthermore, it is important to indicate which lags are more influential. In [8], a lag-specific transfer entropy method was proposed, which evaluates the causality between two time series only based on the informative lags; i.e., the informative delays are selected and the others are discarded.

In this study, we focus on a weather forecasting application as a complex system. Reliable weather forecasting is a central issue since weather conditions can affect our daily life and activities in different ways. State-of-the-art methods make use of Numerical Weather Prediction (NWP), which requires thousands of CPUs for the simulations and consequently is an intensive approach with regards to the computational complexity [9]. In recent years, black-box modeling has been used to address the issue of reliable weather forecasting. Some studies take into account the spatial and temporal properties of the dataset, e.g., Geographically Weighted Regression (GWR) explores the variation of regression coefficients considering spatial information [10]. Some studies have taken advantage of the locality structure of weather conditions to have a better performance. In global learning methods, the same weights are considered for all data points in the training data, while transductive learning algorithms assume that the samples in the test point vicinity are more influential for model fitting [11]. In [12], a clustering-based feature selection for temperature prediction is proposed in which the feature selection and model fitting are done for each cluster independently, and the trained models are used afterwards based on the membership values of the test point to each cluster. In [13], Moving Least Squares Support Vector Machines (M-LSSVM) has been proposed as a soft localization of Least Squares Support Vector Machines (LSSVM) for temperature prediction in Brussels. In this study, we propose a transductive approach for measuring the sample entropy in dynamical systems.

In a data-driven approach, weather forecasting can be seen as a Nonlinear AutoRegressive eXogenous (NARX) model; i.e., the historical data of some nearby cities are taken into account as input features. One may use a feature vector, which is the concatenation of the weather variables from different cities. Taking into account several lags for each variable leads to a high dimensional feature vector. Different studies have deployed information theory to find relevant features in static or dynamic problems [14,15,16,17,18,19]. In this paper, we investigate a global and transductive feature selection for a weather forecasting application. Note that the terms “local” and “global” are considered here in the machine learning sense as in [11] and are not referring to the geographical location of the weather stations. In the global approach, the same weights are considered for all data points in the training data for feature selection, while in the case of the transductive method, the samples in the test point vicinity in feature space are more influential. For the purpose of feature selection, in this study, we deploy the lag-specific information transfer idea to find relevant features in our problem as the globally selected features for the prediction task. In addition, we propose a clustering-based sample entropy methodology, which can be beneficial for transductive feature selection when the local structure of the data is taken into account. In this approach, depending on the clustering information of the training data and the membership values of the test point to the clusters, the samples have different impacts on the sample entropy. Deploying hard clustering can result in using only a part of the training data to measure the sample entropy with the same impact while discarding the other samples. However, using soft clustering, one may exploit the information of the whole dataset while considering different weights for the training samples. In this study, Soft Kernel Spectral Clustering (SKSC) is utilized for the clustering task. Least Squares Support Vector Machines (LSSVM), which is one of the popular learning methods, is used to model the data using the globally- and transductively-selected features.

In this study, the experiments are carried out for temperature prediction in weather forecasting. The data have been collected from the Weather Underground website [20] and include real measurements of weather elements such as temperature, dew point, humidity and wind speed for 10 cities including Brussels, Liege, Antwerp, Amsterdam, Eindhoven, Dortmund, London, Frankfurt, Groningen and Dublin. To evaluate the performance of the proposed method, there are two test sets in different periods of the year: (i) from mid-November 2013 to mid-December 2013 (November/December) and (ii) from mid-April 2014 to mid-May 2014 (April/May). Temperature forecasting is done for both minimum and maximum temperature prediction for 1–6 days ahead in Brussels.

The remaining part of the paper proceeds as follows: first, we explain the background and the proposed method. Then, we present and discuss the results for the application of temperature prediction in weather forecasting, and finally, concluding remarks are presented.

## 2. Materials and Methods

In this section, first we will explain the background of the methodologies that are used as baselines for transductive feature selection using clustering-based sample entropy. Afterwards, the proposed methods will be described in detail.

### 2.1. Background

In this section, we cover the methods used in our algorithm. First, we explain different entropy measures in static and dynamic cases. Secondly, we describe a lag-specific information transfer method, which is used as the main idea of feature selection in our proposed method. Later, Soft Kernel Spectral Clustering (SKSC), which is utilized to find the local structure in our data, and Least Squares Support Vector Machines (LSSVM), which is deployed as a learner, are explained respectively.

#### 2.1.1. Entropy and Information Transfer

Entropy measures are popular methods to investigate the uncertainty of the data. In [21], Shannon discusses that the fundamental problem in a communication system is the reproduction of the message sent from one point to the other point. A communication system includes five elements: (1) the information source, which generates one or more messages to be delivered to the destination; (2) the transmitter, which manipulates the messages to pass them through the channel; (3) the channel, which is a medium to transfer the messages to the destination; (4) the receiver, which retrieves the original message by inverting what the transmitter did; and (5) the destination, which is the intended target of the message.

Shannon defines a measure of uncertainty for the outcome of the system known as Shannon entropy. Given a set of possible events $\Delta =\{\delta_{1},\delta_{2},\ldots ,\delta_{n}\}$ with occurrence probability of $p(\delta_{i})$ for i∈{1,…,n}, the Shannon entropy can be defined as follows:

(1) $$H\left(\Delta \right)=-\sum_{i=1}^{n}p\left(\delta_{i}\right)\log_{2}p(\delta_{i}).$$

In (1), H(Δ) shows the uncertainty in the information that the variable Δ gives about itself. Joint entropy is a measure that evaluates the uncertainty of the outcome when there is more than one random process. Assuming there is another variable Π with a set of possible events $\Pi =\{\pi_{1},\pi_{2},\ldots ,\pi_{n}\}$ and with occurrence probability of $p(\pi_{j})$ for j∈{1,…,n}, the joint entropy can be defined as follows:

(2) $$H\left(\Delta \cap \Pi \right)=-\sum_{j,\;i=1}^{n}p\left(\delta_{i},\pi_{j}\right)\log_{2}p(\delta_{i},\pi_{j}).$$

where $p(\delta_{i},\pi_{j})$ is the probability of the joint occurrence of $\delta_{i}$ and $\pi_{j}$ [21].

Conditional entropy is a measure to assess the uncertainty of a random process while the other one is known. Given the value of Π, the conditional entropy of Δ given Π can be defined as the average of the Shannon entropy as follows [22]:

(3) $$H\left(\Delta |\Pi \right)=\sum_{j=1}^{n}p\left(\pi_{j}\right)H\left(\Delta |\Pi =\pi_{j}\right)=-\sum_{j,\;i=1}^{n}p\left(\delta_{i},\pi_{j}\right)\log_{2}p(\delta_{i}|\pi_{j}).$$

The aforementioned Equations (1)–(3) do not consider the time of the occurrence, and hence, they are known to be static. However, these definitions in information theory play significant roles in the analysis of dynamical systems [23,24,25]. In dynamic processes, the entropy measures can be useful to express the information content of a process over time, e.g., the information that the process is contained at a specific state or the one that is received from the previous states. These methods have been used in a wide range of real-world applications such as climatology, physiology, finance and biology [2,3,4,8,26].

The definition of entropy measures in dynamical processes is similar to the static case. To express the uncertainty in these systems, assume $X_{i}$ indicates a random variable sampled from a dynamic process *X* at time *i*, and $X_{i}^{-}=\left\{X_{1},X_{2},\ldots ,X_{i-1}\right\}$ shows its past states. Given that $p(x_{i})$ is the probability that $X_{i}$ holds the value $x_{i}$ and $S_{i}$ is the set of possible values for $x_{i}$, then the Shannon entropy explains the information content at the current state of the process ($H\left(X_{i}\right)=-\sum_{x_{i}\in S_{i}}p\left(x_{i}\right)\log_{2}p(x_{i})$).

Furthermore, joint entropy expresses the information content of the current and the past states of the random variable *X* as follows:

(4) $$H\left(X_{i}\cap X_{i}^{-}\right)=H\left(X_{i},X_{i}^{-}\right)=-\sum_{x_{j}\in S_{j}}p\left(x_{1},\ldots ,x_{i}\right)\log_{2}p(x_{1},\ldots ,x_{i}).$$

Moreover, conditional entropy is equal to the amount of information that the current state of the random process contained in addition to the past states and can be written as follows:

(5) $$H\left(X_{i}|X_{i}^{-}\right)=H\left(X_{i},X_{i}^{-}\right)-H\left(X_{i}^{-}\right)=-\sum_{x_{j}\in S_{j}}p\left(x_{1},\ldots ,x_{i}\right)\log_{2}p(x_{i}|x_{1},\ldots ,x_{i-1}),$$

where $p(x_{i}|x_{1},\ldots ,x_{i-1})$ is the probability that $X_{i}$ holds the value of $x_{i}$ given that $X_{1}$ to $X_{i-1}$ are $x_{1}$ to $x_{i-1}$, respectively.

In [27], the entropy in dynamical processes is introduced as a predictability measure. Considering that conditional entropy in dynamical processes can be interpreted as new information that can be gained by the current state and is unknown by the previous ones, one may say that if the value of the current state is completely predictable by the previous ones, then there is no new information in the current state; hence, the conditional entropy is equal to zero. Nevertheless, a large conditional entropy shows that the amount of the information generated by the current state is large; thus, there is a lack of information to predict the current state based on its history.

In order to use these measurements in real-world datasets, the experiments rely on the time series prediction. Assume $X=\{x_{1},x_{2},\ldots ,x_{N}\}$ is a time series of length *N*. To deploy the entropy measures, the probability density function can be approximated as follows [28]:

(6) $$p\left(x_{i}\right)=\frac{1}{N}\sum_{j=1}^{N}K(x_{j},x_{i}),$$

where K(·,·) is a kernel function to measure the similarity of $x_{j}$ and $x_{i}$. After having the probability distribution, the Shannon entropy of the time series can be written as follows:

(7) $$H\left(X_{i}\right)=-\ln\left(<p(x_{i})>\right),$$

where $<p(x_{i})>$ indicates the average of $p(x_{i})$ over all possible values $x_{i}$. Furthermore, substituting (7) into (5), the conditional entropy can be expressed in terms of joint probabilities as follows [23]:

(8) $$H\left(X_{i}|X_{i}^{-}\right)=H\left(X_{i},X_{i}^{-}\right)-H\left(X_{i}^{-}\right)=-\ln\left(\frac{<p(x_{1},\ldots ,x_{n-1},x_{n})>}{<p(x_{1},\ldots ,x_{n-1})>}\right).$$

In this study, in order to measure entropy values, we use the special case of the dynamical entropy known as sample entropy [3]. In the sample entropy method, the kernel function K(·,·) is taken to be the Heaviside kernel. The Heaviside kernel sets a threshold *r* on the distance of $x_{i}$ to each sample, i.e., it indicates how many samples are within distance *r* of $x_{i}$. One of the popular approaches is to measure the distance based on the maximum norm, which is the maximum of the absolute difference between each feature of two samples. Considering $\theta \left(x_{i},x_{j}\right)=\max_{1\leq q\leq d}|x_{jq}-x_{iq}|$ where $x_{iq}$ is the *q*-th component (feature) of the *i*-th data point and *d* is the number of features, the Heaviside kernel is expressed as follows:

(9) $$K\left(x_{j},x_{i}\right)=\{\begin{array}{cc} 1, & \theta (x_{i},x_{j})\leq r\\ 0, & \theta \left(x_{i},x_{j}\right)>r. \end{array}$$

Measuring entropy in time series has its own challenges. One important issue in computing entropy criteria is the curse of dimensionality [29]. As the size of the time series becomes larger, the conditional entropy gets closer to zero. Thus, in practice, a limited length of history is taken into account. Considering only *m* previous values for joint probability (to avoid the curse of dimensionality) and excluding the self-match, Equation (8) is equivalent to the sample entropy of the time series [3,23].

Sample entropy can be described as follows: assuming *X* is a realization of a time series $\left\{x_{1},x_{2},\ldots ,x_{N}\right\}$ with length *N*, $X_{i}^{m}$ is a vector of length *m* defined as follows:

(10) $$X_{i}^{m}=\left\{x_{i},x_{i+1},\ldots ,x_{i+m-1}\right\};\;i=1,\ldots ,N-m+1.$$

Excluding the self-match, for *i* ranges from 1 to N−m, $A_{i}^{m}\left(r\right)$ and $B_{i}^{m}\left(r\right)$ in *m*- and (m+1)-dimensional space are calculated as follows:

(11) $$A_{i}^{m}\left(r\right)=\frac{1}{N-m-1}\sum_{\begin{array}{c} j=1\\ j\neq i \end{array}}^{N-m}K(X_{j}^{m},X_{i}^{m})$$

(12) $$B_{i}^{m}\left(r\right)=\frac{1}{N-m-1}\sum_{\begin{array}{c} j=1\\ j\neq i \end{array}}^{N-m}K(X_{j}^{m+1},X_{i}^{m+1}),$$

where K(·,·) is the Heaviside kernel, which is used to indicate how many samples are within distance *r* of $X_{i}^{m}$. Afterwards, $A^{m}\left(r\right)$ and $B^{m}\left(r\right)$ are defined to be equal to the average of $A_{i}^{m}\left(r\right)$ and $B_{i}^{m}\left(r\right)$ over all possible $X_{i}^{m}$:

(13) $$A^{m}\left(r\right)=\frac{1}{N-m}\sum_{i=1}^{N-m}A_{i}^{m}\left(r\right)$$

(14) $$B^{m}\left(r\right)=\frac{1}{N-m}\sum_{i=1}^{N-m}B_{i}^{m}\left(r\right).$$

Finally, the sample entropy in *m*-dimensional space is calculated as follows:

(15) $$SampEnt\left(m,r,N\right)=-\ln\left(\frac{B^{m}\left(r\right)}{A^{m}\left(r\right)}\right).$$

Note that $B^{m}\left(r\right)$ is always smaller than or equal to $A^{m}\left(r\right)$; thus, SampEnt(m,r,N) has a non-negative value.

As previously explained, the entropy measures can be interpreted as a predictability power. In this study, we deploy the sample entropy definition to find relevant delays in a NARX model. In this scheme, in *m* dimensions (refer to (11)), the predictor time series are presented and in the m+1 dimension (refer to (12)), and the target time series is added to them.

#### 2.1.2. Lag-Specific Information Transfer

As previously mentioned, entropy criteria can be utilized to determine how much information is transferred from the previous states of a dynamical process to the current one. This can be extended to investigate more than one dynamical process and evaluate their relations and influences on each other. The authors in [8] have proposed a lag-specific transfer entropy method to evaluate the information transfer. Given $X_{n}$ and $Y_{n}$ are the values of time series *X* and *Y* at time *n*, $X_{n}^{-}=\left\{X_{n-1},X_{n-2},\ldots \right\}$ and $Y_{n}^{-}=\left\{Y_{n-1},Y_{n-2},\ldots \right\}$ indicate the past values of the time series, based on the Granger causality (G-causality), there is a G-causality from *X* to *Y* if $X_{n}^{-}$ includes the information that can improve the prediction of $Y_{n}$ above and beyond the information that $Y_{n}^{-}$ involves [30]. The amount of the information contained in $X_{n}^{-}$ can be measured using the following:

(16) $$I\left(Y_{n};X_{n}^{-}|Y_{n}^{-}\right)=H\left(Y_{n}|Y_{n}^{-}\right)-H\left(Y_{n}|X_{n}^{-},Y_{n}^{-}\right).$$

Based on the definition, there is G-causality from $X_{n}^{-}$ to *Y* if and only if $I(Y_{n};X_{n}^{-}|Y_{n}^{-})>0$ [31].

The authors in [8] have discussed the fact that this approach generally accumulates the G-causal influence of all past values, and therefore, it does not consider the lag-specific information; i.e., the amount of information that specific state $X_{n-t}$ gives to $X_{n}$ is unknown. In order to make it lag-specific, an itemized approach is proposed: there is G-causality from $X_{n-t}$ to *Y* if $X_{n-t}$ includes information that can improve the prediction of $Y_{n}$ above and beyond the information that both $Y_{n}^{-}$ and $X_{n}^{-}\backslash X_{n-t}$ involve. Note that $X_{n}^{-}\backslash X_{n-t}=\left\{X_{n-1},\ldots ,X_{n-t-1},X_{n-t+1},\ldots \right\}$. This approach can exploit the amount of information in each lag of different dynamical processes, and therefore, the informative lags can be identified. Eventually, the transfer entropy can only aggregate the information contained in the informative past values. The procedure of finding informative past values can be described as follows.

Assuming *V* as a set of selected influential and informative components and $V^{\prime }$ as a set of candidate components, then $V\cap V^{\prime }=\emptyset$ and $V\cup V^{\prime }=\left\{X_{n-1},\ldots ,X_{n-L_{max}},Y_{n-1},\ldots ,Y_{n-L_{max}}\right\}$ where $L_{max}$ is the maximum lag to be taken into account. Note that in this study, $X_{i}$ and $Y_{i}$ for i∈{1,…,n} are uni-variate time series. The procedure of detecting influential lags is an iterative procedure where $V_{k}$ and $V_{k}^{\prime }$ indicate *V* and $V^{\prime }$ at iteration *k*, respectively. The algorithm starts with $V_{0}$ as an empty set. In each iteration, for each $W\in V_{k-1}^{\prime }$, a candidate set $\left\{W,V_{k-1}\right\}$ is created, and the conditional entropy $H(Y_{n}|W,V_{k-1})$ is computed. The component *W* that results in the minimum conditional entropy $\left(\arg\min_{W}H(Y_{n}|W,V_{k-1})\right)$ is selected, and subsequently, *V* and $V^{\prime }$ are updated as follows: $V_{k}=\left\{W,V_{k-1}\right\}$ and $V_{k}^{\prime }=V_{k-1}^{\prime }\backslash W_{k}$. The procedure terminates when an irrelevant component is added to the selected set *V*.

The relevance of the selected component is evaluated based on the significance of the reduction in the conditional entropy as follows:

(17) $$I\left(Y_{n};W_{k}|V_{k-1}\right)=H\left(Y_{n}|V_{k-1}\right)-H\left(Y_{n}|V_{k}\right).$$

To determine the significance of the reduction in the conditional entropy, a statistical approach is used. The statistical significance is estimated by deploying time shifted surrogate data [8]. In this approach, the surrogate data are generated by multiple shifting of the original series $W_{k}$ for randomly-selected lag with respect to $Y_{n}$. For example, assuming $W_{k}$ has *N* elements and $W_{k}=\left[W_{k1},W_{k2},\ldots ,W_{kN}\right]$, then the shifted time series with lag equal to *l* is $\left[W_{k(l+1)},W_{k(l+2)},\ldots ,W_{kN},W_{k1},\ldots ,W_{kl}\right]$ [32]. Afterwards, the reduction of the conditional entropy is evaluated for the original series and the new shifted ones. If the reduction of the conditional entropy for the original one is below the 100(1–*a*) percentile of its distribution on the surrogate data, $W_{k}$ is considered to be an irrelevant feature (delay), and the termination condition is fulfilled; otherwise, $W_{k}$ is a relevant variable and is added to the selected set *V*. Note that the lag variable for shifting $W_{k}$ has to be large enough (not close to one and *N*) to eliminate the causality effect between the new shifted time series and the output. If the null hypothesis is rejected, one can be sure that the reduction in the conditional entropy is in fact because of causality and not random.

The authors in [8] have used the idea of relevant component selection to evaluate the transfer entropy between the variables and measuring the amount of the information that they transfer to each other. Nevertheless, in this paper, regardless of the amount of the transferred information, we use the lag-specific component selection idea as a forward feature selection approach to find relevant features in an NARX model. Therefore, if a lag-specific component contains information based on the (17), then it is selected as a relevant feature.

#### 2.1.3. Soft Kernel Spectral Clustering

To take advantage of the local structure of the data, one may use clustering. As previously said, using soft clustering can be beneficial to exploit the knowledge of all samples while considering different weights for the samples in each cluster depending on the membership of the test point to that cluster. In this study, we use Soft Kernel Spectral Clustering (SKSC), which is one of the popular non-linear clustering methods [33].

Assume κ is the number of clusters and $x_{i}$ is a row vector including *d* features for i∈{1,2,…,N}. Considering *l* as the number of score variables needed to encode the κ clusters, the projection of the training data in the feature space can be represented by $e^{\left(l\right)}={\left[e_{1}^{\left(l\right)},\ldots ,e_{N}^{\left(l\right)}\right]}^{T}$. Let $\gamma_{l}\in R^{+}$ be the regularization parameter. The SKSC primal formulation is expressed as follows [33,34]:

(18) $$\begin{array}{cc} \min_{w^{\left(l\right)},b_{l},e^{\left(l\right)}} & \frac{1}{2}\sum_{l=1}^{\kappa -1}{w^{\left(l\right)}}^{T}w^{\left(l\right)}-\frac{1}{2N}\sum_{l=1}^{\kappa -1}\gamma_{l}{e^{\left(l\right)}}^{T}D_{\Omega }^{-1}e^{\left(l\right)}\\ \mathrm{subject}\;\;\mathrm{to} & e^{\left(l\right)}=\Phi w^{\left(l\right)}+b_{l}1_{N},\;l=1,\ldots ,\kappa -1. \end{array}$$

Here, $\varphi \left(\cdot \right):R^{d}\to R^{h}$ is the feature map that maps the data to a high or infinite dimensional space and $\Phi =[\varphi {\left(x_{1}\right)}^{T},\ldots ,\varphi {\left(x_{N}\right)}^{T}]$ is a N×h matrix. Ω is the kernel matrix where $\Omega_{ij}=K\left(x_{i},x_{j}\right)$, and Mercer’s theorem [35] can be expressed as follows:

(19) $$\Omega_{ij}=\varphi {\left(x_{i}\right)}^{T}\varphi \left(x_{j}\right)=K\left(x_{i},x_{j}\right)\;\;\;i,j=1,2,\ldots ,N.$$

Note that for positive definite kernel function K(·,·), one may exploit Mercer’s theorem to implicitly use the feature map, and thus, φ(·) does not have to be explicitly defined.

In addition, $D_{\Omega }\in R^{N\times N}$ is the diagonal degree matrix associated with the Ω where $D_{\Omega }^{\left(i,i\right)}=\sum_{j}\Omega_{ij}$.

Let $\alpha^{\left(l\right)}\in R$ be the Lagrange multipliers. Then, based on the Lagrangian $L(w\left(l,b_{l},e^{\left(l\right)};\alpha^{\left(l\right)}\right)=\frac{1}{2}\sum_{l=1}^{\kappa -1}{w^{\left(l\right)}}^{T}w^{\left(l\right)}-\frac{1}{2N}\sum_{l=1}^{\kappa -1}\gamma_{l}{e^{\left(l\right)}}^{T}D_{\Omega }^{-1}e^{\left(l\right)}-\sum_{l=1}^{\kappa -1}\alpha^{\left(l\right)}\left(e^{\left(l\right)}-\left(\Phi w^{\left(l\right)}+b_{l}1_{N}\right)\right)$, the optimality conditions for l=1,…,κ−1 are as follows [34]:

(20) $$\left\{\begin{array}{c} \frac{\partial L}{\partial w^{\left(l\right)}}=0\to w^{\left(l\right)}=\Phi^{T}\alpha^{\left(l\right)}\\ \frac{\partial L}{\partial b_{l}}=0\to 1_{N}^{T}\alpha^{\left(l\right)}=0\\ \frac{\partial L}{\partial e^{\left(l\right)}}=0\to \alpha^{\left(l\right)}=\frac{\gamma_{l}}{N}D_{\Omega }^{-1}e^{\left(l\right)},\\ \frac{\partial L}{\partial \alpha^{\left(l\right)}}=0\to e^{\left(l\right)}=\Phi w^{\left(l\right)}+b_{l}1_{N}. \end{array}\right.$$

After eliminating $w^{\left(l\right)}$, $b_{l}$ and $e^{\left(l\right)}$, the dual problem is described as follows:

(21) $$\begin{array}{c} D_{\Omega }^{-1}M_{D}\Omega \alpha^{\left(l\right)}=\lambda_{l}\alpha^{\left(l\right)} \end{array}$$

where $\alpha^{\left(l\right)}$ is the vector of dual variables, $\lambda_{l}=\frac{N}{\gamma_{l}}$, and $M_{D}=I_{N}-\left(1/1_{N}^{T}D_{\Omega }^{-1}1_{N}\right)\left(1_{N}1_{N}^{T}D_{\Omega }^{-1}\right)$ is a centering matrix.

The clustering models in dual space for a given sample $x_{i}$ is as follows:

(22) $$\begin{array}{c} e_{i}^{\left(l\right)}=\sum_{j=1}^{N}\alpha_{j}^{\left(l\right)}K\left(x_{j},x_{i}\right)+b_{l},\;l=1,\ldots ,\kappa -1,j=1,\ldots ,N. \end{array}$$

After finding the initial borders of clusters, the prototypes in the score variables’ space are recalculated to improve the clusters’ borders, and subsequently, the data points are assigned to a cluster based on their distance to the prototypes. The prototype $\psi_{c}$ of cluster *c* for c=1,…,κ can be found as follows:

(23) $$\psi_{c}=\frac{1}{N_{c}}\sum_{i=1}^{N_{c}}e_{i}^{\left(l\right)},$$

where $N_{c}$ is the number of samples in cluster *c* and $e_{i}^{\left(l\right)}$ are the score variables of the samples in cluster *c*.

In this study, we use the Radial Basis Kernel (RBF) (24) as the kernel function; thus, the kernel bandwidth σ together with the number of clusters κ are the two parameters that have to be tuned. The RBF kernel is:

(24) $$K\left(x_{i},x_{j}\right)=\exp(-||x_{i}-x_{j}|{|}_{2}^{2}/\sigma^{2}).$$

In this study, Average Membership Strength (AMS) is employed to tune the hyperparameters based on the grid search, and the values that yield the maximum AMS are selected. Thus, for different numbers of clusters (in this study, from 2–10) and different values of the kernel bandwidth, AMS on the validation set is evaluated, and the one that has the maximum AMS is selected. In AMS, the average membership value for the samples in the validation set to each cluster is calculated based on the cosine similarities between the samples and the prototypes of the corresponding cluster.

For a given test point $x_{test}$, the membership value to the cluster *c* is expressed as follows [33]:

(25) $$Memb_{test}^{\left(c\right)}=\frac{\prod_{j\neq c}d_{test,j}^{cos}}{\sum_{h=1}^{\kappa }\prod_{j\neq h}d_{test,j}^{cos}},\sum_{h=1}^{\kappa }Memb_{test}^{\left(h\right)}=1,$$

where κ is the number of cluster and $d_{test,j}^{cos}$ is the cosine similarity between the test sample and the prototype of the cluster *j* in the score variables space.

#### 2.1.4. Least Squares Support Vector Machines

Least Squares Support Vector Machines (LSSVM) is a well-known machine learning method proposed in [36,37]. The main difference between Support Vector Machines (SVM) and LSSVM is the fact that instead of quadratic programming in SVM, LSSVM solves a set of linear equations. Let $x\in R^{d}$, y∈R and $\varphi :R^{d}\to R^{h}$ where φ(·) is a feature map and *h* is the dimension of the feature map. The model in primal space is formulated as follows:

(26) $$y\left(x\right)=w^{T}\varphi \left(x\right)+b$$

where b∈R and $w\in R^{h}$. The optimization problem is written as follows [37]:

(27) $$\begin{array}{cc} \min_{w,b,e} & \frac{1}{2}w^{T}w+\frac{\gamma }{2}\sum_{j=1}^{N}e_{j}^{2}\\ \mathrm{subject}\;\;\mathrm{to} & y_{j}=w^{T}\varphi \left(x_{j}\right)+b+e_{j},j=1,\ldots ,N, \end{array}$$

where ${\left\{x_{j},y_{j}\right\}}_{j=1}^{N}$ is the training set, $\gamma \in R^{+}$ is the regularization parameter and $e_{j}=y_{j}-{\hat{y}}_{j}$ is the error between the actual and predicted output for data point *j*.

Let $\alpha_{j}\in R$ be the Lagrange multipliers. Then, based on the Lagrangian $L\left(w,b,e;\alpha \right)=\frac{1}{2}w^{T}w+\frac{\gamma }{2}\sum_{j=1}^{N}e_{j}^{2}-\sum_{j=1}^{N}\alpha_{j}\left(w^{T}\varphi \left(x_{j}\right)+b+e_{j}-y_{j}\right)$, the optimality conditions are as follows:

(28) $$\left\{\begin{array}{c} \frac{\partial L}{\partial w}=0\to w=\sum_{j=1}^{N}\alpha_{j}\varphi \left(x_{j}\right)\\ \frac{\partial L}{\partial b}=0\to \sum_{j=1}^{N}\alpha_{j}=0\\ \frac{\partial L}{\partial e_{j}}=0\to \alpha_{j}=\gamma e_{j},j=1,\ldots ,N\\ \frac{\partial L}{\partial \alpha_{j}}=0\to y_{j}=w^{T}\varphi \left(x_{j}\right)+b+e_{j},j=1,\ldots ,N. \end{array}\right.$$

After eliminating *w* and *e*, the dual problem can be formulated as follows:

(29) $$\left(\begin{array}{cc} 0 & 1_{N}^{T}\\ 1_{N} & \Omega +\frac{1}{\gamma }I_{N} \end{array}\right)\left(\frac{b}{\alpha }\right)=\left(\frac{0}{y}\right),$$

where Ω is the kernel matrix. In this study, we deploy RBF as a kernel function, which is formulated in (24). Thus, the regularization parameter γ and the kernel parameter σ are the tuning parameters.

Having $\alpha_{j}$ and *b* as the solution for the linear system, the LSSVM model as a function estimator is expressed as follows:

(30) $$\hat{y}\left(x\right)=\sum_{j=1}^{N}\alpha_{j}K\left(x,x_{j}\right)+b.$$

In this study, we use LSSVM to learn the model based on the selected features; thus, good performance can be an indication that relevant features have been selected.

### 2.2. Transductive Feature Selection Using Clustering-Based Sample Entropy

In this study, we propose a methodology for transductive feature selection based on the clustering-based sample entropy. The seasonal behavior of the weather condition is the intuition to investigate the transductive feature selection. Mostly, feature selection methods take into account the relevance of the features for prediction in the whole dataset. However, in the transductive feature selection, we assume that some features can be considered relevant in some part of the data while being irrelevant when all samples are taken into account and vice versa. In [12], a clustering-based feature selection is deployed in weather forecasting. It is shown that selecting features based on the clustering information can result in a better performance for weather prediction.

Given that weather forecasting can be seen as a Nonlinear AutoRegressive eXogenous (NARX) model [12,38] and assuming $Y_{t}$ and $X_{p,t}$ for p=1,…,d are the output and *p*-th exogenous inputs of the system at time *t* and *s* is a positive integer denoting the number of steps ahead in the future to predict, the NARX model can be written as follows:

(31) $${\hat{Y}}_{t+s}=f\left(Y_{t},Y_{t-1},\ldots ,Y_{t-L_{max}},X_{1,t},X_{1,t-1},\ldots ,X_{1,t-L_{max}},\ldots ,X_{d,t},X_{d,t-1},\ldots ,X_{d,t-L_{max}}\right)$$

Having $X\in R^{N\times d}$ and $Y\in R^{N}$, where $X_{j,i}$ is the value of the exogenous input *j* at time *i* and $Y_{i}$ is the value of the uni-variate time series *Y* at time *i*, we define $X_{train}=\left[X_{1:d,1:N-L_{max}},X_{1:d,2:N-L_{max}+1},\ldots ,X_{1:d,L_{max}:N-1},Y_{1:N-L_{max}},\ldots ,Y_{L_{max}:N-1}\right]$ and $X_{test}=\left[X_{1:d,N-L_{max}+1},X_{1:d,N-L_{max}+2},\ldots ,X_{1:d,N},Y_{N-L_{max}+1:N}\right]$. Note that $X_{train}\in R^{\left(N-L_{max}\right)\times \left(\left(d+1\right)\times L_{max}\right)}$ and $X_{test}\in R^{1\times (\left(d+1\right)\times L_{max})}$.

**Figure 1:**
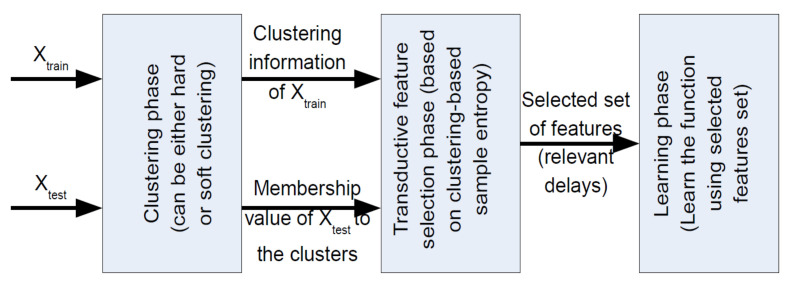
The flowchart of transductive feature selection.

The diagram of the proposed method is depicted in Figure 1. As is shown, the algorithm consists of three main steps. In the first block, a clustering algorithm is applied on the data. The output of this block includes the clustering information of the training samples and the membership of the test point to each cluster. Depending on the membership values of the test point, some parts of the data-set are considered for the feature selection. Using hard clustering, only a subset of the data, which includes the samples of the cluster that the test point belongs to, is passed to the next block to find relevant features. However, using soft clustering, all data points can be used in the next block depending on the membership values of the test point to the clusters. In this study, we deploy SKSC in the clustering block. Thus, we exploit the information of all data: the data in each cluster affect the feature selection procedure based on the membership of the test point to the corresponding cluster.

**Figure 2:**
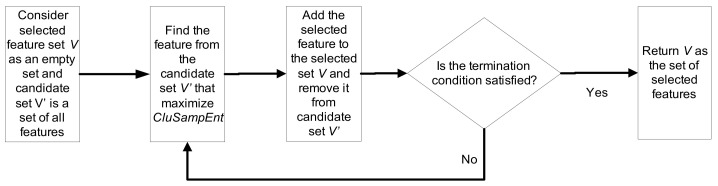
Feature selection using clustering-based sample entropy.

In the second block, the feature selection procedure is defined by finding informative lags or delays of the input time series. Knowing the samples in each cluster and the membership values of the test point to each cluster, the feature selection using the clustering-based sample entropy is an iterative procedure that has been shown in Figure 2 and can be expressed as follows.

Let *V* be the set of selected informative lags of the time series and $V^{\prime }$ be the candidate components; thus, similar to lag-specific transfer entropy, $V\cap V^{\prime }=\emptyset$ and $V\cup V^{\prime }=\left\{X_{1,1:N-L_{max}},\ldots ,X_{1,L_{max}:N-1},\ldots ,X_{d,1:N-L_{max}},\ldots ,X_{d,L_{max}:N-1},Y_{1:N-L_{max}},\ldots ,Y_{L_{max}:N-1}\right\}$ where $X_{i,t_{1}:t_{2}}$ is a column vector including the values of the time series $X_{i}$ in the time period of $t_{1}$ to $t_{2}$. The feature selection is done in an iterative procedure where $V_{k}$ and $V_{k}^{\prime }$ indicate *V* and $V^{\prime }$ at iteration *k*, respectively. The algorithm starts with $V_{0}$ as an empty set. Each iteration can be explained in three steps:

1. For each $W\in V_{k-1}^{\prime }$, a candidate set $\left\{W,V_{k-1}\right\}$ is created, and the conditional entropy $H(Y_{n}|W,V_{k-1})$ is computed based on the clustering-based sample entropy.
2. The component *W* that minimizes the conditional entropy $\left(\arg\min_{W}H(Y_{n}|W,V_{k-1})\right)$ is selected to be added to the selected set *V*.
3. *V* and $V^{\prime }$ are updated as follows: $V_{k}=\left\{W,V_{k-1}\right\}$ and $V_{k}^{\prime }=V_{k-1}^{\prime }\backslash W_{k}$, and the termination condition is checked.

The procedure terminates when an irrelevant component is added to the selected set *V*. In this study, we utilize the surrogate data to evaluate the relevance of the selected feature as explained in Section 2.1.2.

The clustering-based sample entropy in iteration *k* can be expressed as follows:

1. Assuming $S_{k}\in R^{N\times k}$ is the concatenation of the selected set of features $V_{k}$ for all samples, the samples can be partitioned into separated groups based on the clustering information such that $S_{k}^{c}\in R^{N_{c}\times k}$ represents the selected features for the samples in the cluster *c*.
2. In each cluster, for *i* ranging from 1–$N_{c}$, $A_{i}^{k,c}\left(r\right)$ and $B_{i}^{k,c}\left(r\right)$ in *k* and k+1 dimensional space are calculated as follows:
  (32) $$A_{i}^{k,c}\left(r\right)=\frac{1}{N-1}\sum_{\begin{array}{c} j=1\\ j\neq i \end{array}}^{N_{c}}K(S_{j,k}^{c},S_{i,k}^{c}),$$
  (33) $$B_{i}^{k,c}\left(r\right)=\frac{1}{N-1}\sum_{\begin{array}{c} j=1\\ j\neq i \end{array}}^{N_{c}}K(\left[S_{j,k}^{c},Y_{j}^{c}\right],\left[S_{i,k}^{c},Y_{i}^{c}\right]),$$
  where K(·,·) is the Heaviside kernel which is used to indicate how many samples are within distance *r* of $S_{i,k}^{c}$. In this step, the probability density function is calculated for two cases: using only selected features and based on selected features together with the target value. Therefore, the conditional entropy of the target value given selected features can be calculated. Note that, $\sum_{c}A_{i}^{k,c}\left(r\right)=A_{i}^{k}\left(r\right)$ and $\sum_{c}B_{i}^{k,c}\left(r\right)=B_{i}^{k}\left(r\right)$ where $A_{i}^{k}\left(r\right)$ and $B_{i}^{k}\left(r\right)$ are equivalent to (11) and (12) in the sample entropy definition.
3. Similar to sample entropy, $A^{k,c}\left(r\right)$ and $B^{k,c}\left(r\right)$ in *k* and k+1 are defined to be equal to the average of $A_{i}^{k,c}\left(r\right)$ and $B_{i}^{k,c}\left(r\right)$ over all possible $V_{i,k}^{c}$:
  (34) $$A^{k,c}\left(r\right)=\frac{1}{N}\sum_{i=1}^{N}A_{i}^{k,c}\left(r\right),$$
  (35) $$B^{k,c}\left(r\right)=\frac{1}{N}\sum_{i=1}^{N}B_{i}^{k,c}\left(r\right).$$
  Note that $\sum_{c}A^{k,c}\left(r\right)=A^{k}\left(r\right)$ and $\sum_{c}B^{k,c}\left(r\right)=B^{k}\left(r\right)$ where $A^{k}\left(r\right)$ and $A^{k}\left(r\right)$ are equivalent to (13) and (14) in sample entropy definition.
4. Finally, the clustering-based sample entropy (CluSampEnt), which represents the conditional entropy, in *k* dimensional space is calculated as follows:
  (36) $$CluSampEnt\left(k,r,N\right)=-\ln\left(\frac{\sum_{c}Memb_{test}^{\left(c\right)}B^{k,c}\left(r\right)}{\sum_{c}Memb_{test}^{\left(c\right)}A^{k,c}\left(r\right)}\right)$$
  where $Memb_{test}^{\left(c\right)}$ is the membership value of the test point to cluster *c*. Note that $B^{k,c}\left(r\right)$ is always smaller than or equal to $A^{k,c}\left(r\right)$; thus, CluSampEnt(k,r,N) has a non-negative value.

Clustering-based sample entropy can be considered as a transductive entropy measure as it gives us more information about the samples that are more similar to the given test point. Note that if the membership values of the test point to all clusters are equal, the clustering-based sample entropy is equivalent to the sample entropy; thus, all training data points have the same influence on the conditional entropy.

Finally, in the last block in Figure 1, a learner is used to model the data using the selected features. In this study, we use LSSVM for learning the data based on the selected features. A better performance on prediction indicates that more relevant features have been selected.

## 3. Results

### 3.1. Experiments on the Simulated Dataset

In this section, we have deployed the proposed methods to find the relevant delays of variables in linear and nonlinear synthetic datasets. In addition, we have compared the proposed method with three other methodologies. The first one is Automatic Relevance Determination (ARD), which is a popular feature selection approach in a Bayesian framework. We have used the implementation of ARD in the framework of LSSVM (LSSVM Toolbox Version 1.8, KU Leuven, Leuven, Belgium) [39]. This method involves three levels of inference: in the first level, the model parameters (the primal weights and bias) are estimated based on the prior, which corresponds to the sum of the squared error and the regularization parameters; in the second level, the hyperparameters, which are utilized to avoid over-fitting and under-fitting, are estimated; and in the third level, the kernel parameter estimation and the model comparison are done [37]. The second approach deploys partial conditioning based on Mutual Information (MI) as an entropic measure. In this method, to select the first feature, the mutual information of each feature with the target value is evaluated, and the one that leads to the maximum value is added to the selected set. In the next iterations, the feature that jointly with the previously selected features has the maximum mutual information with the target value is added to the selected set. The procedure continues until a pre-defined number of features is selected [18]. Finally, we utilized Least Absolute Shrinkage and Selection Operator (LASSO), which is a popular feature selection approach proposed by [40]. LASSO is a regularization method that produces sparse models by imposing an L1-norm penalty on the regression coefficients. Note that both proposed methods and the MI-based method are model-free approaches, while ARD and LASSO are model-based methods.

We have created 10 realizations of all systems for 1000 time steps and with random initialization. To evaluate the performance of different methods for a linear system, consider the following system:

(37) $$\begin{array}{c} u_{t}=0.9u_{t-1}-0.6u_{t-2}-2.1+e_{u,t},\;\;\;for\;\;\;1\leq t\leq 1000\\ y_{t}=0.7y_{t-1}+0.8u_{t-3}+1.8+e_{y,t},\;\;\;for\;\;\;1\leq t\leq 1000, \end{array}$$

where $e_{u,t}$ and $e_{y,t}$ are independent white noise with zero mean and 0.5 variance. As was previously mentioned, in this paper, we assume that data in different clusters are a function of different variables. Therefore, we define the localized linear system example as follows:

(38) $$\begin{array}{cc} Cluster1(1\leq t\leq 500): & u_{t}=0.9u_{t-1}-0.6u_{t-2}+e_{u,t},\\ & y_{t}=0.7y_{t-1}+0.8u_{t-3}+e_{y,t}\\ Cluster2(501\leq t\leq 1000): & u_{t}=0.9u_{t-1}-0.6u_{t-2}+e_{u,t}\\ & y_{t}=0.81y_{t-2}+0.95u_{t-4}+e_{y,t}. \end{array}$$

Note that in the first 500 points, $y_{t}$ is a function of $y_{t-1}$ and $u_{t-3}$, while in the next 500 points, it is related to $y_{t-2}$ and $u_{t-4}$.

In the rest of the paper, we refer to Systems (37) and (38) as the global and localized linear system, respectively. Similar to the linear systems, consider a nonlinear global system defined as follows:

(39) $$\begin{array}{c} u_{t}=3.4u_{t-1}\left(1-u_{t-1}^{2}\right)exp\left(-u_{t-1}^{2}\right)+e_{u,t},\;\;\;for\;\;\;1\leq t\leq 1000\\ y_{t}=3.4y_{t-2}\left(1-y_{t-2}^{2}\right)exp\left(-y_{t-2}^{2}\right)+1.5u_{t-1}^{2}+e_{y,t},\;\;\;for\;\;\;1\leq t\leq 1000. \end{array}$$

We have made some changes in the global system such that the underlying processes for the first and the second 500 time steps are different such that the localized nonlinear system is defined as follows:

(40) $$\begin{array}{cc} Cluster1(1\leq t\leq 500): & u_{t}=3.4u_{t-1}\left(1-u_{t-1}^{2}\right)exp\left(-u_{t-1}^{2}\right)+e_{u,t},\\ & y_{t}=3.4y_{t-1}\left(1-y_{t-1}^{2}\right)exp\left(-y_{t-1}^{2}\right)+3.9u_{t-3}^{2}+e_{y,t}.\\ Cluster2(501\leq t\leq 1000): & u_{t}=1.4u_{t-1}\left(1-u_{t-1}^{2}\right)exp\left(-u_{t-1}^{2}\right)+e_{u,t},\\ & y_{t}=4.4y_{t-2}\left(1-y_{t-2}^{2}\right)exp\left(-y_{t-2}^{2}\right)-3.9u_{t-1}^{2}+e_{y,t}. \end{array}$$

Note that in the first 500 points, $y_{t}$ is a function of $y_{t-1}$ and $u_{t-3}$, while in the next 500 points, it is related to $y_{t-2}$ and $u_{t-1}$.

Considering $L_{max}=5$ and r=0.1, the occurrence of the first two relevant features in both global and transductive feature selections together with ARD, MI-based and LASSO approaches are shown in Table 1. As is shown, all methods perform equally well for the global linear system, and they are competitive in the case of the global nonlinear system. Note that in all cases, the most relevant features for the prediction of $y_{t}$ are selected.

In Table 2 and Table 3, the occurrence of the first two selected features for two test samples with different membership values to the clusters in the localized systems are presented; that is, Table 2 shows the occurrence when the test point membership values to the first and second cluster are 0.8 and 0.2, respectively; thus, the dependency of variables in the test point is closer to the first cluster underlying model. However, in Table 3, the membership values to the clusters are 0.2 and 0.8; so, the pattern in the test point is closer to the second cluster underlying model. The results reveal that in this scenario, the proposed transductive feature selection approach outperforms other approaches as it selects the relevant features more times. This is expected as other methods select the features based on considering that all data points have the same effect.

In the rest of the experiment part, we evaluate the performance of the proposed global and transductive feature selection approaches on the application of weather forecasting.

### 3.2. Weather Dataset

**Figure 3:**
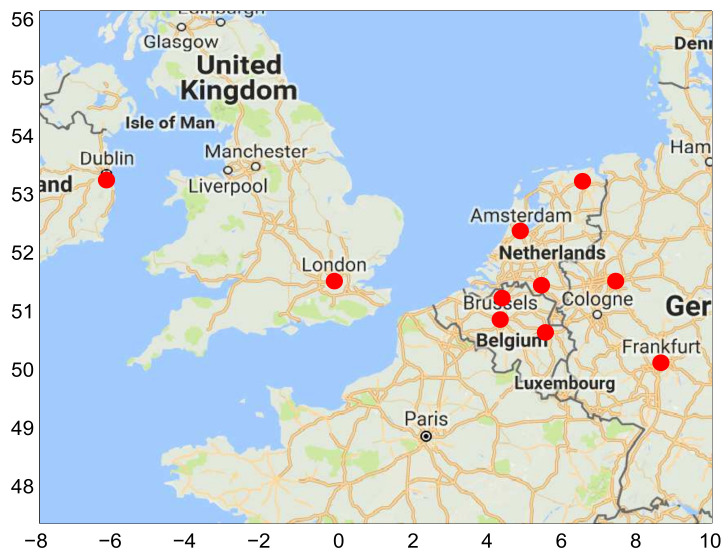
Weather data stations.

In this study, data have been gathered from the Weather Underground website and include real measurements of weather variables such as minimum and maximum temperature, dew point, humidity and wind speed for 10 cities including Brussels, Antwerp, Liege, Amsterdam, Eindhoven, Dortmund, London, Frankfurt, Groningen and Dublin, as shown in Figure 3.

In order to assess the performance of the proposed methods in different weather conditions, the performance is reported on two test sets in different time periods: (i) from mid-November 2013–mid-December 2013 (November/December) and (ii) from mid-April 2014–mid-May 2014 (April/May).

The data cover a time period from the beginning of 2007–mid-2014 and contain 180 measured weather variables for each day. Note that the number of training samples is different for each test data point, and it is based on the number of days from the beginning of 2007 until the day before the test date.

### 3.3. Weather Forecasting Experiments

In this study, the experiments are done for minimum and maximum temperature prediction in Brussels for 1–6 days ahead. For both SKSC and LSSVM, the dual problem is implemented, and there are different parameters that need to be tuned: the number of clusters and the kernel bandwidth parameter in SKCS are tuned using AMS considering 60% of data for training and 40% for validation; furthermore, the regularization parameter and the kernel bandwidth in LSSVM are tuned using 10-fold cross-validation. In this study, we consider $L_{max}$ to be 10 and generate 50 realizations of the shifted surrogate data. Three different values [0.7,1,1.6] have been considered for the *r* value in the Heaviside kernel, and the results are reported for all of them.

**Figure 4:**
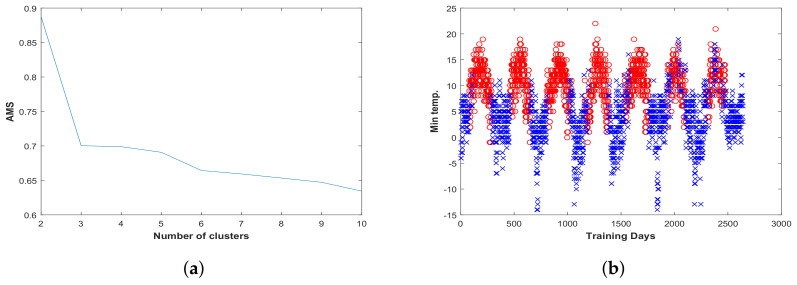
(**a**) Average Membership Strength (AMS) value for different numbers of clusters; (**b**) clustered training data.

Figure 4 shows the AMS value for different numbers of clusters, and it can be seen that the maximum value of AMS is when the data are divided into two clusters. In addition, the clusters have been depicted in Figure 4, and one may say that the clusters indicate different patterns in different periods of a year. Note that having one cluster means that the same weights are considered for all data points, and hence, it is equivalent to the global feature selection approach.

In order to compare the accuracy of the data-driven approaches with the state-of-the-art methods in weather forecasting, the performances of the black-box methods are compared with the one of the Weather Underground company. In addition, we utilize the global and transductive feature selection to identify relevant delays of each weather variable. Note that there are 180 weather variables in the dataset, and for each of them, we consider $L_{max}$ to be 10. In this case, there are 1800 features, which is equal to $180\times L_{max}$. Furthermore, we compare the results with the one when there is no feature selection.

In Table 4 and Table 5, the average Mean Absolute Error (MAE) of the prediction using the global and transductive feature selection for the minimum and maximum temperature in two test sets are compared. For each value of *r*, the method that has a better performance is bolded. In most of the cases, transductive feature selection results in a lower MAE, which can be an indication that selecting features transductively is able to identify relevant features better than the global approach.

**Figure 5:**
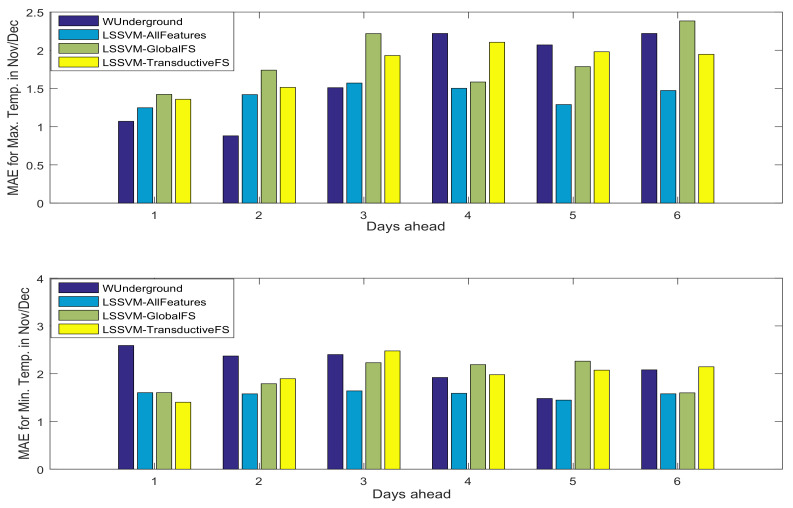
Mean absolute Error (MAE) of maximum (**top**) and minimum (**bottom**) temperature prediction in the test set November/December. LSSVM: Least Squares Support Vector Machines.

**Figure 6:**
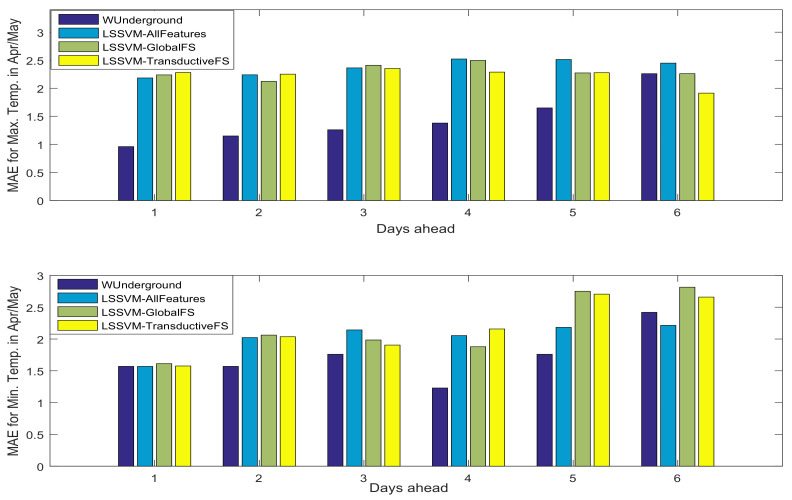
MAE of maximum (**top**) and minimum (**bottom**) temperature prediction in the test set April/May.

In Figure 5 and Figure 6, the performance of the Weather Underground prediction is compared with the ones of the black-box methods for both test sets. The black-box approaches utilized LSSVM when: (1) there is no feature selection; (2) global feature selection is deployed; and (3) transductive feature selection is used. Note that in the case of utilizing feature selection, the *r* value is tuned using cross-validation. As is shown, the data-driven approaches are competitive with the state-of-the-art methods in weather forecasting. In the test set November/December, the black-box methods outperform Weather Underground, while Weather Underground shows more reliable weather forecasting in April/May. This can be due to the lower variance in observations in the test set November/December. In addition, the performances of the black-box methods when feature selection methods are employed are competitive with the case that there is no feature selection.

**Figure 7:**
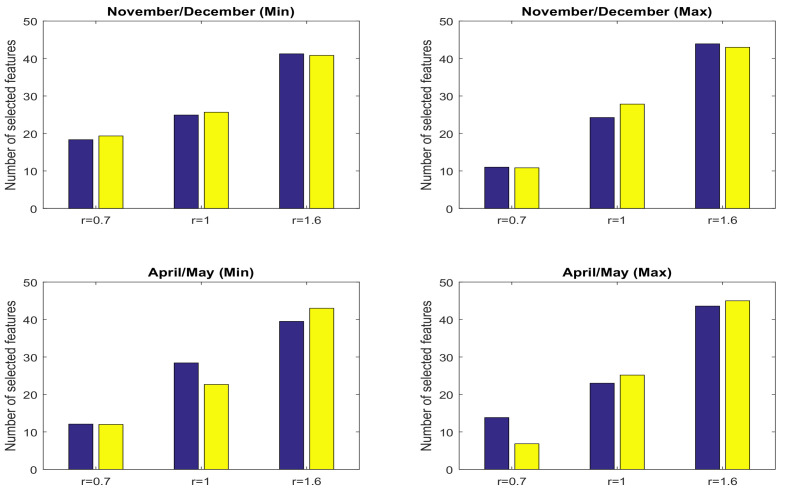
Average number of selected features for the November/December and April/May test set for minimum (**left**) and maximum (**right**) temperature using different *r* values.

In order to analyze the reduction in the complexity of the methods in terms of the number of features, Figure 7 depicts the average number of selected features for different values of *r*. Obviously, increasing the value of *r* results in a larger number of selected features. This phenomenon is expected as with a larger *r* value, it takes more iterations for the conditional entropy to decay to zero; thus, more features are selected at the end of the feature selection procedure.

As was mentioned, there are 180 weather variables, and considering $L_{max}$ to be 10, the total number of features is 1800. Figure 7 suggests that in all cases, there is more than a 97% reduction in the number of features. Taking Figure 5 and Figure 6 into account, it can be seen that although there is a huge reduction in the number of features, the results are competitive with the case of using all features. In addition, we have investigated the prediction intervals as described in [41], and we have observed that even though we are reducing the number of features significantly, the prediction intervals are competitive, which indicates that the level of uncertainty is not increased.

**Figure 8:**
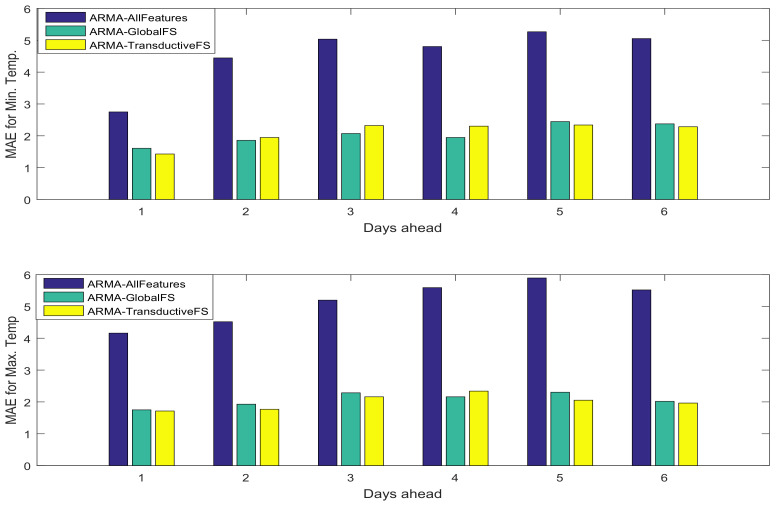
Mean Absolute Error (MAE) of minimum (**top**) and maximum (**bottom**) temperature prediction of the AutoRegressive with eXogenous input (ARX) model in both test sets.

In addition to LSSVM as an NARX machine learning approach, we have investigated the impact of the feature selection on a linear approach such as the AutoRegressive with eXogenous input (ARX) model. The overall performances of prediction on both test sets together with using the ARX model in three cases (1) without feature selection, (2) features selected by the global approach and (3) features selected by the transductive approach are presented in Figure 8. As is shown, the feature selection methods can improve the performance of the linear models significantly even though very few features are selected.

**Figure 9:**
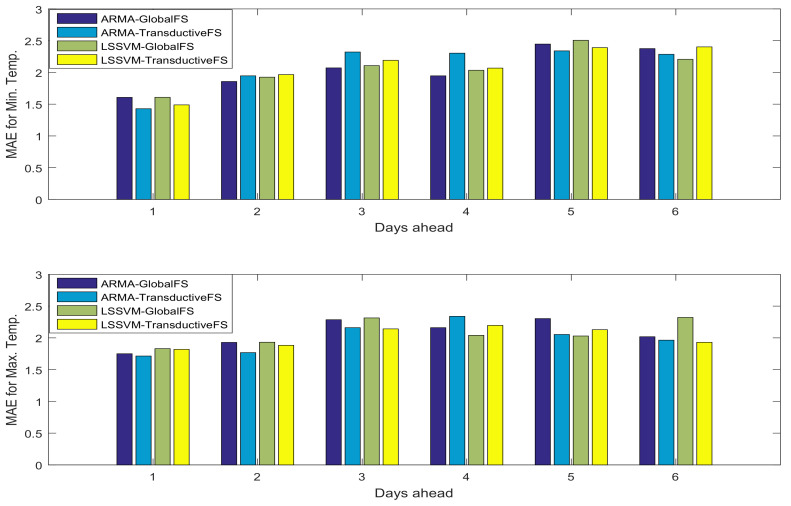
MAE of minimum (**top**) and maximum (**bottom**) temperature prediction of the ARX and LSSVM models while the proposed feature selection methods are deployed in both test sets.

The comparison between the performance of the linear model (ARX) and the nonlinear model (LSSVM), while the proposed feature selection methods are used, is depicted in Figure 9. As is shown, the performances of both models are competitive, and this may be due to the efficient feature selection approach.

**Figure 10:**
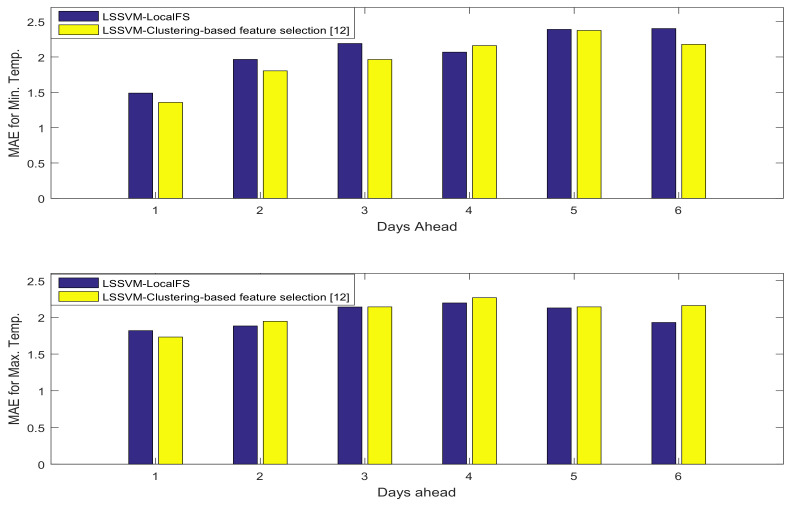
MAE of minimum (**top**) and maximum (**bottom**) temperature prediction of the proposed transductive feature selection and the method in [12] in both test sets.

Moreover, since the proposed method benefits from the clustering information to find informative features, we have compared the results on the weather dataset with the one proposed in [12], which also deploys clustering information to improve the feature selection performance. The mean absolute error of the minimum and maximum temperature prediction is shown in Figure 10. The experimental results reveal that while the number of selected features using the method in [12] is three to four-times larger than the number of features selected by the proposed transductive feature selection approach, the performances of both methods are competitive.

Note that there are some major differences between the proposed method and the one in [12]. While the proposed transductive feature selection method in this study is model-free, the one in [12] is a linear feature selection approach. In addition, in the proposed methods, depending on the membership values of each test point, the selected features can be different, while in [12], the features are selected per cluster, and the membership values of the test point affect the prediction.

**Figure 11:**
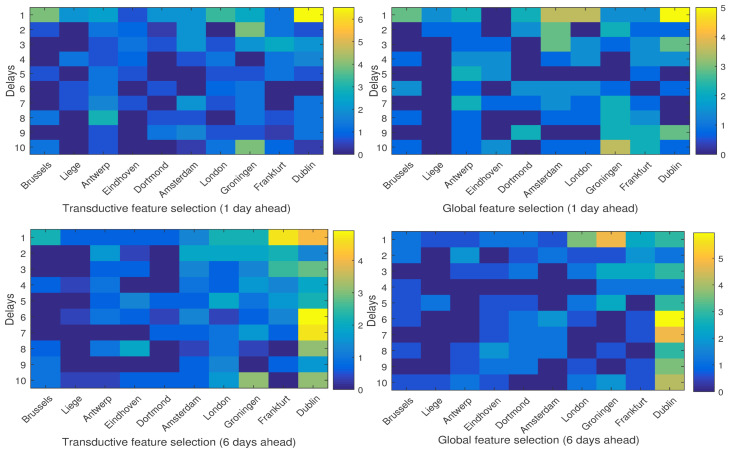
The average percentage of the selected features per delay in each city in the test set November/December.

**Figure 12:**
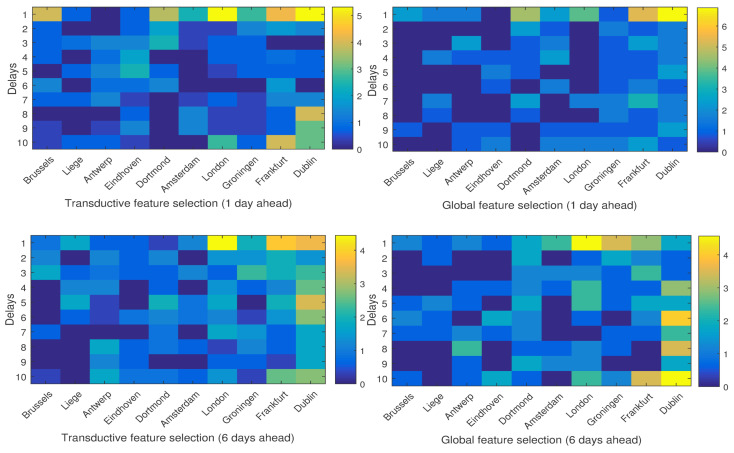
The average percentage of the selected features per delay in each city in the test set April/May.

In order to investigate the influence of each neighboring city on the temperature of Brussels, Figure 11 and Figure 12 show the percentage of the selected features from different cities and different delays. As is depicted, in both global and local feature selection, for short-term prediction (one day ahead), closer cities such as Brussels itself, Dortmond and Amsterdam seem more relevant, while for long-term prediction (six days ahead), farther cities such as Dublin, London and Groningen are more important. Moreover, in short-term prediction, short histories (smaller delays) are more relevant, while for the long-term one, larger delays should also be taken into account.

## 4. Discussion

In this study, we propose a feature selection approach based on the entropy measures in an application of weather forecasting. The sample entropy was used to measure the conditional entropy of the target value, which is the maximum and minimum temperature in Brussels, while a set of features, which includes weather variables, is given. This set was formed in a forward selection of the time series that are affecting the target time series. The influence of time series on each other is measured using the conditional entropy; i.e., smaller conditional entropy shows a higher power of predictability.

The results suggest that selecting the informative time series that are affecting the target value can reduce the complexity of the model in terms of the number of features significantly. However, the performance of the model remains good even when there is a smaller number of variables in the model. Surprisingly, in many cases, the performance is improved. In addition, a smaller number of features can be beneficial for the visualization task in a complex network such as climate data.

The results of these experiments support the conclusion of the paper [12] in which it is shown that taking into account the local structure of data can result in better performance in weather forecasting. In addition, it is depicted that in different periods of the year, which means different weather conditions, the influence of the neighboring cities on the weather variables of the target city can be different. For example, in Figure 11 and Figure 12, in the case of six-day-ahead prediction, London seems more influential in the April/May test set, while in the November/December test set, Frankfort is more informative.

A major drawback of the proposed transductive feature selection method, which uses the clustering-based sample entropy, is the fact that for each test point the whole procedure should be done independently. In daily weather forecasting, in each day, there is only one test point for which the weather conditions for 1–6 days ahead should be predicted. Considering the fact that in this application, the trained model should be updated on a daily basis, the transductive approach does not have higher complexity than the global one. However, in some datasets with more than one test point, the transductive feature selection becomes time consuming. One possible solution for this problem can be clustering the test points. Since the test points in each cluster are similar to each other, their membership values to the clusters in the training data should be similar, as well. Therefore, transductive feature selection can be done for each cluster of the test points independently. Note that the proposed method is applicable for any time series prediction, such as climate, financial or medical systems, since it investigates the impact of regressor time series on the target one.

## 5. Conclusions

In this study, we investigated a feature selection approach based on entropy measures in an application of weather forecasting. We deployed the sample entropy to evaluate the conditional entropy of the target value when a set of selected features is given. The forward selection approach is followed; thus, in each iteration, the variable that minimizes the conditional entropy was added to the set of selected features. In addition, considering the local structure of the data, we proposed the clustering-based sample entropy, which is similar to the sample entropy definition except the fact that the clustering information of the training data and the membership of the test point to the clusters are taken into account to perform the feature selection.

The performances of black-box methods are compared with the one of the Weather Underground company, and the experiments show that the data-driven weather forecasting is competitive with the state-of-the-art methods in this field. The results reveal that utilizing the proposed feature selection methodologies leads to a significant decrease in the number of features, while the performance remains adequate. Moreover, the experiments suggest that the transductive feature selection can improve the performance of finding relevant variables.

## Figures and Tables

**Table 1:** Number of times that the corresponding feature is selected using 10 different initial values (test point in the global system (37), (39)).

Method	Linear System										Nonlinear System									
	$u_{t-1}$	$y_{t-1}$	$u_{t-2}$	$y_{t-2}$	$u_{t-3}$	$y_{t-3}$	$u_{t-4}$	$y_{t-4}$	$u_{t-5}$	$y_{t-5}$	$u_{t-1}$	$y_{t-1}$	$u_{t-2}$	$y_{t-2}$	$u_{t-3}$	$y_{t-3}$	$u_{t-4}$	$y_{t-4}$	$x_{t-5}$	$y_{t-5}$
Global-FS	0	10	0	0	10	0	0	0	0	0	10	0	0	10	0	0	0	0	0	0
Transductive-FS	0	10	0	0	10	0	0	0	0	0	10	0	0	10	0	0	0	0	0	0
ARD [37]	0	10	0	0	10	0	0	0	0	0	10	0	0	10	0	0	0	0	0	0
MI-based [18]	0	10	0	0	10	0	0	0	0	0	10	4	1	2	1	0	0	2	0	0
LASSO [40]	0	10	0	0	10	0	0	0	0	0	10	3	0	1	2	0	0	6	0	0

FS: Feature Selection; ARD: Automatic Relevance Determination; MI: Mutual Information; LASSO: Least Absolute Shrinkage and Selection Operator.

**Table 2:** Number of times that the corresponding feature is selected using 10 different initial values (test point in the localized systems (38), (40): membership values to clusters [0.8, 0.2]).

Method	Linear System										Nonlinear System									
	$u_{t-1}$	$y_{t-1}$	$u_{t-2}$	$y_{t-2}$	$u_{t-3}$	$y_{t-3}$	$u_{t-4}$	$y_{t-4}$	$u_{t-5}$	$y_{t-5}$	$u_{t-1}$	$y_{t-1}$	$u_{t-2}$	$y_{t-2}$	$u_{t-3}$	$y_{t-3}$	$u_{t-4}$	$y_{t-4}$	$u_{t-5}$	$y_{t-5}$
Global-FS	0	9	0	2	7	0	1	1	0	0	8	1	0	9	2	0	0	0	0	0
Transductive-FS	0	9	0	0	10	1	0	0	0	0	0	10	0	0	9	0	0	1	0	0
ARD [37]	0	0	0	10	0	0	10	0	0	0	1	0	0	9	10	0	0	0	0	0
MI-based [18]	0	5	0	7	3	0	5	0	0	0	1	0	0	9	0	7	0	1	0	2
LASSO [40]	0	10	0	10	0	0	0	0	0	0	0	0	0	10	6	1	0	0	0	3

**Table 3:** Number of times for which the corresponding feature is selected using 10 different initial values (test point in the localized system (38), (40): membership values to clusters [0.2, 0.8]).

Method	Linear System										Nonlinear System									
	$u_{t-1}$	$y_{t-1}$	$u_{t-2}$	$y_{t-2}$	$u_{t-3}$	$y_{t-3}$	$u_{t-4}$	$y_{t-4}$	$u_{t-5}$	$y_{t-5}$	$u_{t-1}$	$y_{t-1}$	$u_{t-2}$	$y_{t-2}$	$u_{t-3}$	$y_{t-3}$	$u_{t-4}$	$y_{t-4}$	$u_{t-5}$	$y_{t-5}$
Global-FS	0	9	0	2	7	0	1	1	0	0	8	1	0	9	2	0	0	0	0	0
Transductive-FS	0	0	0	10	0	0	10	0	0	0	10	0	0	10	0	0	0	0	0	0
ARD [37]	0	0	0	10	0	0	10	0	0	0	1	0	0	9	10	0	0	0	0	0
MI-based [18]	0	5	0	7	3	0	5	0	0	0	1	0	0	9	0	7	0	1	0	2
LASSO [40]	0	10	0	10	0	0	0	0	0	0	0	0	0	10	6	1	0	0	0	3

**Table 4:** Mean Absolute Error (MAE) for minimum and maximum temperature prediction in November/December.

Step Ahead	Temp.	*r* = 0.7		*r* = 1		*r* = 1.6	
		Global-FS	Transductive-FS	Global-FS	Transductive-FS	Global-FS	Transductive-FS
1	Min	**1.48 ± 0.001**	1.54 ± 0.001	1.52 ± 0.001	**1.45 ± 0.004**	1.68 ± 0.001	**1.50 ± 0.001**
	Max	1.76 ± 0.001	**1.73 ± 0.003**	**1.42 ± 0.001**	1.47 ± 0.003	1.45 ± 0.003	**1.39 ± 0.001**
2	Min	2.15 ± 0.0001	**1.95 ± 0.004**	1.98 ± 0.001	**1.77 ± 0.01**	**1.76 ± 0.001**	1.89 ± 0.001
	Max	2.13 ± 0.001	**1.72 ± 0.002**	1.88 ± 0.003	**1.80 ± 0.001**	1.73 ± 0.003	**1.49 ± 0.02**
3	Min	2.07 ± 0.005	**2.00 ± 0.003**	**1.90 ± 0.001**	1.98 ± 0.01	**2.16 ± 0.001**	2.33 ± 0.004
	Max	**1.77 ± 0.002**	1.88 ± 0.03	**2.13 ± 0.001**	2.33 ± 0.2	2.14 ± 0.001	**1.90 ± 0.003**
4	Min	**1.59 ± 0.003**	1.80 ± 0.002	2.21 ± 0.001	**2.05 ± 0.01**	2.22 ± 0.001	**1.96 ± 0.02**
	Max	2.37 ± 0.001	**2.25 ± 0.001**	2.18 ± 0.003	**2.15 ± 0.001**	**1.54 ± 0.002**	2.06 ± 0.001
5	Min	2.37 ± 0.001	**2.21 ± 0.001**	**2.20 ± 0.001**	2.25 ± 0.001	2.46 ± 0.001	**2.29 ± 0.004**
	Max	2.19 ± 0.001	**1.94 ± 0.01**	**1.92 ± 0.001**	2.29 ± 0.2	**1.79 ± 0.001**	**1.89 ± 0.05**
6	Min	2.40 ± 0.006	**2.31 ± 0.005**	**1.66 ± 0.001**	2.19 ± 0.02	**2.17 ± 0.001**	2.30 ± 0.1
	Max	1.95 ± 0.001	**1.93 ± 0.002**	2.42 ± 0.001	**1.82 ± 0.005**	2.36 ± 0.004	**1.71 ± 0.01**

**Table 5:** MAE for minimum and maximum temperature prediction in April/May.

Step Ahead	Temp.	*r*= 0.7		*r* = 1		*r* = 1.6	
		Global-FS	Transductive-FS	Global-FS	Transductive-FS	Global-FS	Transductive-FS
1	Min	1.65 ± 0.001	**1.59 ± 0.001**	1.74 ± 0.001	**1.46 ± 0.001**	1.63 ± 0.001	**1.53 ± 0.001**
	Max	2.09 ± 0.001	**2.04 ± 0.001**	**2.23 ± 0.001**	**2.23 ± 0.001**	2.31 ± 0.001	**2.18 ± 0.003**
2	Min	**2.01 ± 0.001**	2.20 ± 0.002	2.09 ± 0.001	**1.98 ± 0.002**	2.06 ± 0.001	**1.98 ± 0.002**
	Max	2.31 ± 0.001	**2.18 ± 0.005**	**2.09 ± 0.001**	2.29 ± 0.002	**2.12 ± 0.001**	2.25 ± 0.001
3	Min	**2.11 ± 0.001**	2.29 ± 0.004	2.27 ± 0.001	**2.03 ± 0.002**	**2.12 ± 0.001**	**2.12 ± 0.01**
	Max	2.52 ± 0.001	**2.48 ± 0.004**	2.83 ± 0.002	**2.56 ± 0.001**	2.47 ± 0.001	**2.40 ± 0.002**
4	Min	3.01 ± 0.001	**2.69 ± 0.001**	**2.59 ± 0.004**	2.63 ± 0.001	**2.01 ± 0.001**	2.25 ± 0.003
	Max	2.39 ± 0.004	**2.10 ± 0.001**	**2.32 ± 0.004**	2.42 ± 0.03	2.49 ± 0.001	**2.28 ± 0.003**
5	Min	**2.90 ± 0.001**	2.98 ± 0.002	2.50 ± 0.001	**2.40 ± 0.002**	2.87 ± 0.002	**2.80 ± 0.001**
	Max	2.56 ± 0.004	**2.39 ± 0.001**	2.62 ± 0.005	**2.54 ± 0.005**	**2.27 ± 0.001**	2.37 ± 0.001
6	Min	2.74 ± 0.003	**2.59 ± 0.001**	**2.66 ± 0.001**	2.70 ± 0.004	2.80 ± 0.001	**2.57 ± 0.001**
	Max	**2.25 ± 0.02**	2.35 ± 0.001	**1.96 ± 0.002**	2.64 ± 0.008	2.26 ± 0.005	**1.91 ± 0.002**
